# Non-Contrast Brain CT Images Segmentation Enhancement: Lightweight Pre-Processing Model for Ultra-Early Ischemic Lesion Recognition and Segmentation

**DOI:** 10.3390/jimaging11100359

**Published:** 2025-10-13

**Authors:** Aleksei Samarin, Alexander Savelev, Aleksei Toropov, Aleksandra Dozortseva, Egor Kotenko, Artem Nazarenko, Alexander Motyko, Galiya Narova, Elena Mikhailova, Valentin Malykh

**Affiliations:** 1 Higher School of Digital Culture, ITMO University, St. Petersburg 197101, Russia; 2 Faculty of Radio Engineering and Telecommunications, St. Petersburg Electrotechnical University “LETI”, St. Petersburg 197022, Russia; 3 Department of Microbiological Synthesis Technology, St. Petersburg State Institute of Technology, St. Petersburg 190013, Russia; 4 Mathematics and Mechanics Faculty, St. Petersburg State University, St. Petersburg 198504, Russia; 5 Information Systems Department, International IT University, Almaty 050000, Kazakhstan

**Keywords:** image preprocessing, ischemic stroke recognition, computer tomography snapshots, image segmentation

## Abstract

Timely identification and accurate delineation of ultra-early ischemic stroke lesions in non-contrast computed tomography (CT) scans of the human brain are of paramount importance for prompt medical intervention and improved patient outcomes. In this study, we propose a deep learning-driven methodology specifically designed for segmenting ultra-early ischemic regions, with a particular emphasis on both the ischemic core and the surrounding penumbra during the initial stages of stroke progression. We introduce a lightweight preprocessing model based on convolutional filtering techniques, which enhances image clarity while preserving the structural integrity of medical scans, a critical factor when detecting subtle signs of ultra-early ischemic strokes. Unlike conventional preprocessing methods that directly modify the image and may introduce artifacts or distortions, our approach ensures the absence of neural network-induced artifacts, which is especially crucial for accurate diagnosis and segmentation of ultra-early ischemic lesions. The model employs predefined differentiable filters with trainable parameters, allowing for artifact-free and precision-enhanced image refinement tailored to the challenges of ultra-early stroke detection. In addition, we incorporated into the combined preprocessing pipeline a newly proposed trainable linear combination of pretrained image filters, a concept first introduced in this study. For model training and evaluation, we utilize a publicly available dataset of acute ischemic stroke cases, focusing on the subset relevant to ultra-early stroke manifestations, which contains annotated non-contrast CT brain scans from 112 patients. The proposed model demonstrates high segmentation accuracy for ultra-early ischemic regions, surpassing existing methodologies across key performance metrics. The results have been rigorously validated on test subsets from the dataset, confirming the effectiveness of our approach in supporting the early-stage diagnosis and treatment planning for ultra-early ischemic strokes.

## 1. Introduction

In the domain of contemporary medicine, strokes and other consequences of ischemic attacks represent one of the primary causes of mortality and disability worldwide. According to the World Health Organization (WHO), approximately 15 million individuals suffer from strokes annually, with 5 million resulting in fatal outcomes and another 5 million leading to long-term disability [1]. The increasing prevalence of ischemic strokes is closely correlated with the rise in risk factors such as hypertension, diabetes mellitus, obesity, and sedentary lifestyles. Despite advancements in medical technologies and treatment methodologies, the long-term repercussions of strokes continue to exert a profound impact on public health and the quality of life for millions globally [2].

The prevention of strokes and the early detection of ischemic attacks constitute critical aspects of modern medical diagnostics. Magnetic Resonance Imaging (MRI) [3] plays a pivotal role in this process, providing high-resolution cross-sectional images of the brain, which enable the identification and quantification of ischemic regions. Additionally, non-contrast computed tomography (NCCT) [4] is widely employed. Early stroke detection is particularly crucial, as it facilitates the precise delineation of ischemic zones and initiates immediate medical intervention and treatment. However, the manual interpretation of medical imaging by specialists presents a formidable challenge due to the subtle and intricate nature of ischemic lesions [5], which are often difficult to discern with the naked eye. This underscores the necessity of leveraging advanced computational methodologies to accurately identify and analyze affected regions.

To address these challenges, modern technologies, particularly deep neural networks (DNNs) [6,7,8,9,10,11,12,13,14,15,16,17,18,19], have emerged as a promising solution. These models have demonstrated high performance in solving computer vision tasks, including image segmentation, which is crucial in automating the detection of ischemic regions. Image segmentation involves mapping a pixel-wise mask of the lesion area onto the original medical image, thereby automating the detection process and significantly improving accuracy and efficiency.

In this study, we focus on the task of segmenting ischemic regions in non-contrast computed tomography (NCCT) scans [4]. Our approach addresses two critical segmentation tasks:

- Segmentation of the Ischemic Penumbra [20]. This region of the ischemic lesion includes brain tissues and cells that have the potential to recover with medical intervention.
- Segmentation of the Ischemic Core [21]. This area consists of cells that have irreversibly lost their function.

By accurately segmenting both the penumbra and the core, our method provides a comprehensive analysis of the ischemic regions, which is crucial for effective treatment planning. Automating ischemia region segmentation requires high-quality segmentation models. Furthermore, in industrial settings, cost reduction remains a critical factor, necessitating the development of computationally efficient models.

Therefore, we focus on developing a lightweight image preprocessing approach to enhance the segmentation quality of the ischemic penumbra and ischemic core region segmentation. Preprocessing models have recently been widely adopted in medical applications and have proven their effectiveness [22,23]. The proposed model effectively mitigates artifacts such as low contrast, high/low brightness, blurring, and noise, thereby improving image quality and, consequently, the accuracy of image segmentation models. Artifacts in CT scans [24,25] arise from various factors, including the limited contrast range of soft tissues, patient motion artifacts, interpolation artifacts, and other hardware and software limitations of this imaging technique [26]. The use of contrast agents can improve diagnostic accuracy and partially mitigate artifacts; however, this method is more expensive, has several contraindications, and requires a prolonged acquisition time, which is unacceptable in cases of ischemic stroke, where every second is critical.

The development of generative computer vision (including GAN-based models, diffusion models, and autoencoders) has also influenced the task of improving image color enhancement. Existing state-of-the-art solutions are typically end-to-end architectures [27,28,29,30,31] and tend to be computationally heavy. The direct application of generative approaches without additional processing is unsuitable for medical diagnostic tasks, as such models are prone to generating various neural network artifacts and may fabricate details that are not present in the original image.

A promising approach to improving image quality is the use of combined neural network methods based on filters [29,32,33,34,35,36], which integrate neural networks for parameter generation with classical mathematical differentiable functions for modeling various filters, such as contrast adjustment, sharpening, white balance correction, and others.

Our proposed model employs a combined neural network approach that addresses these limitations. It is more robust and incapable of generating artifacts, as it does not directly modify the image but instead predicts parameters for predefined differentiable filters.

We introduce a lightweight combined image preprocessing model for ischemia region segmentation. To evaluate the effectiveness of the proposed preprocessing model, we conducted a series of experiments that compared the quality of the segmentation of ischemic regions before and after applying our approach. We used several key metrics, such as IoU 3D and Dice 3D, to assess the impact of preprocessing on segmentation outcomes objectively. As a result, our model demonstrated a significant improvement in segmentation accuracy.

Within the scope of our study, we developed a novel lightweight, trainable neural network block composed of image filters that enhance the effectiveness of biomedical image processing tasks within the preprocessing pipeline proposed in this work. The suggested approach is based on a learnable linear combination of convolutions with varying kernel sizes, employing pretrained filter kernels. These kernels were trained using an encoder-decoder framework on a subset of the employed dataset. This innovation enables improved performance of the overall preprocessing pipeline, offering an efficient solution for enhancing image quality and feature representation in biomedical imaging applications.

Our approach significantly enhances the accuracy of identifying critical health issues in patients. It facilitates precise and timely intervention for managing the consequences of ischemic attacks and enables the implementation of preventive measures to avert strokes and other adverse outcomes. The improved recognition and segmentation capabilities contribute to better patient outcomes and more effective healthcare interventions, potentially reducing the incidence and severity of stroke-related disabilities.

The contributions of this work can be summarized as follows:

- We propose a lightweight combined neural network approach for preprocessing non-contrast computer tomography (NCCT) brain images to enhance the segmentation quality of the ischemic core and penumbra regions.
- We also introduce a lightweight, trainable image filtering module based on a learnable linear combination of convolutions with pretrained kernels of multiple sizes, integrated into a biomedical image preprocessing pipeline to enhance task performance.
- As the baseline architecture for the preprocessing model, we use the LFIEM/UNIFI model [32,33], with minor modifications to its architecture and image processing filters.
- Our approach demonstrates improved performance on the CPAISD dataset [37] (extemely hard case), combined RSNA-MICCAI [38] and CQ500 [39] (as a general case) in terms of IoU 3D and Dice 3D metrics using various segmentation pipelines.

## 2. Problem Statement

**Figure 1:**
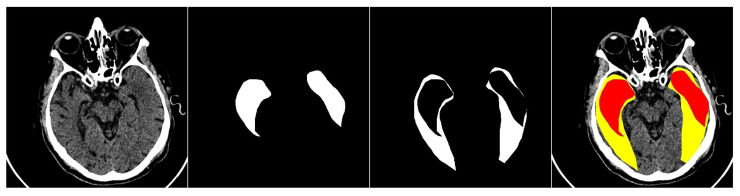
An example of an input image with three classes. The first one is the original CT scan, the second is the ischemic core mask, the third is the penumbra area mask, and the fourth is the visualization with mask overlay (ischemic core in red, penumbra area in yellow).

This study primarily focuses on enhancing the performance of multiclass segmentation in brain CT images. The input image is segmented into three regions: ischemic core, penumbra area, and background. The segmentation model produces three output masks, each corresponding to a specific region, with the label “1” representing a significant region and “0” otherwise (Figure 1).

## 3. Materials and Methods

**Figure 2:**
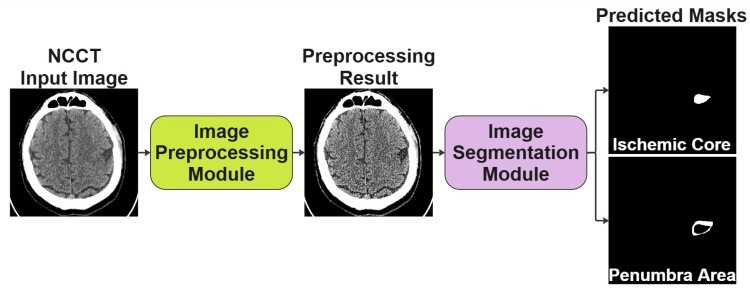
The conceptual scheme of the two-stage solution we proposed for segmenting NCCT scans of the human brain.

The conceptually developed solution is a two-stage process and consists of an Image Preprocessing Module and an Image Segmentation Module, as shown in Figure 2. The preprocessing block is necessary to improve the original image, while the segmentation block follows to segment the image and generate the prediction masks, respectively. In the following subsections, we discuss each block in more detail and the specifics of its joint training.

### 3.1. Image Preprocessing Module

Our methodology is based on the theoretical framework outlined in the reference works [32,33,34,40,41], which were specifically selected for their strong alignment with the objectives of our study. The LFIEM/UNIFI preprocessing stack was chosen due to its lightweight architecture (45 K parameters are negligible numbers of relatively widely used methods for biomedical image segmentation: U-Net—7.8 M, U-Net++—9.0 M, Attention U-Net—8.5–9.2 M, ResUNet—15–17 M, ResUNet++—18–20 M, nnU-Net—30–40 M, V-Net—3.5 M, 3D U-Net—19–25 M, DeepLabV3+ ResNet-50—40–50 M, TransUNet—105 M, Swin UNETR—60–62 M, UNETR—90–96 M, MedT—28–30 M, SegFormer MiT-B0 to B5—14–47 M, nnFormer—60–80 M, UXNet—30–35 M, nnU-Net 3D FullRes—38–45 M, DynUNet—25–50 M, CARUNet—22–25 M, EMANet—10–12 M, Dense U-Net/DenseVNet—5–8 M, MultiResUNet—10–11 M, H-Net/HyperDense-Net—2–4 M, MA-Net—30–35 M, PraNet—30 M, CoTr—65–70 M, MISSFormer—60 M, PMFS-Net—35 M, CASCADE—45–50 M, BiO-Net—12–15 M), computational efficiency, and deterministic behavior, critical requirements for clinical deployment. Unlike end-to-end deep learning approaches, which often require extensive parameter optimization and may introduce unpredictable artifacts, our method relies on carefully curated filter combinations with fixed, interpretable operations. This design ensures minimal computational overhead (reducing hardware constraints for integration into medical imaging systems) while maintaining robust performance. Importantly, the absence of trainable nonlinear transformations in the preprocessing stage eliminates the risk of hallucinated features or adversarial artifacts, a crucial consideration for diagnostic reliability. The stack’s modularity also allows for targeted adjustments to specific imaging modalities without compromising stability, making it particularly suitable for heterogeneous clinical environments where reproducibility and transparency are paramount. The models described in these works were carefully designed as standalone solutions for enhancing image color gamut; however, in some of the studies, they are also applied as preprocessing modules for addressing various tasks.

We choose the LFIEM model [32,33] as the baseline model to enhance the original frame. We modify this model by replacing multiple copies of the parameter generator with a single generator that simultaneously produces parameters for all applied filters. Additionally, we employ different enhancement filters that are suitable for our problem formulation.

Although non-contrast CT (NCCT) images are inherently single-channel data represented in Hounsfield Units (HU), our preprocessing pipeline operates in RGB space for principled architectural and empirical reasons. First, the input HU values are rigorously preprocessed using clinically relevant brain windowing (WW = 80, WL = 40) and linearly normalized to the [0, 1] range to preserve tissue contrast critical for early ischemic lesion detection. This normalized single-channel image is then duplicated across the R, G, and B channels to form a 3-channel tensor—not to introduce color information, but to conform to the expected input dimensionality of modern deep learning backbones (including our parameter generator and downstream segmenters like SwinUNet and UNet++), which are overwhelmingly optimized for 3-channel inputs due to their ImageNet pretraining heritage. This design choice is not arbitrary: in controlled ablation experiments (Section 4.4.2), the RGB-space configuration consistently outperformed its 1-channel HU-space counterpart by +3.1% Dice on the challenging CPAISD dataset and +1.8% on the mixed RSNA-MICCAI+CQ500 benchmark. We attribute this gain to improved gradient flow, better feature reuse, and framework-level optimizations for 3D tensor operations in PyTorch 1.7.1. Critically, since all three RGB channels are identical copies and all filters operate channel-wise with shared parameters, no artificial chromatic information is introduced—the “RGB” representation is purely a tensor shape convention. Final outputs are clipped to [0, 1], which is safe and consistent because all transformations operate within this normalized dynamic range. Thus, our use of RGB space is a lightweight, artifact-free, performance-enhancing adaptation, not a distortion of the underlying medical signal, and represents a pragmatic alignment of clinical data with modern deep learning infrastructure.

**Figure 3:**
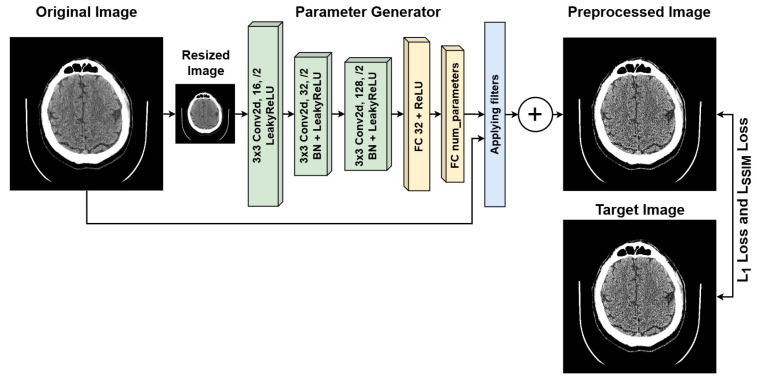
Architecture of the proposed lightweight image preprocessing module.

The schematic of our proposed approach is presented in Figure 3. The preprocessing module is lightweight and consists of three convolutional blocks [42] and two fully connected layers. It operates on a downscaled copy of the image, as the applied transformations remain consistent across different scales of the same image. At the output of the parameter generator, an activation function is used. Depending on the required range, this is the sigmoid function [43] for the range [0,1], the hyperbolic tangent function [44] for the range [−1,1], or no activation function [45] at all if the corresponding transformation does not require it. The filters with the parameters obtained are then applied to the original image, and the resulting image is obtained by summing the outputs of all the applied filters, followed by clipping the values into the range [0,1]. We also explored the applicability of other transformations, including attentional transformations [46]. Just as we do not use additional resolution enhancement modules [47] for reasons of saving computing resources and reducing hardware requirements. However, our parameter encoder is extremely lightweight and thus suitable only for predicting the trainable coefficients of linear combinations, which ensures the lightweight nature of the architecture. Despite the exclusion of projection transformations, which are necessary for supporting methods such as attention-based ones, the efficiency of our preprocessing is quite significant given the lightweight nature of the model.

In addition to the basic installation of the optimal filter configuration, we have added an additional custom block specially developed within the main preprocessing stack to improve the quality of segmentation. We will describe the structure and setup procedure for this block below.

Let us introduce some notations. Let $I_{o}$ be the original image, $I_{so}$ its small version, *h* the parameter generator for the filters, and $I_{e}$ the enhanced version of the image, $f_{i}$ denotes corresponding filter transformation. Then, the described solution can also be expressed in the form of the following general formula:

(1) $$p_{1..n}=h\left(I_{so}\right),$$

(2) $$I_{e}=min\left(max\left(I_{o}+\sum_{i=1}^{n}f_{i}\left(I_{o},p_{i}\right),0\right),1\right),$$

where the functions min and max are used to show the clipping into the range [0,1], as we work in the RGB space.

The research area examined in this study is highly specific; therefore, we propose a preprocessing procedure for CT scan images using the filters listed below. A series of experiments was conducted to achieve optimal results for combinations of two and three filters. The results of these experiments are presented in the Section 4. It is worth noting that we consider the filters in the context of their application in the RGB space.

Thus, we consider the following differentiable transformations.

**Gaussian blur filter.** This is a local convolution operation that uses a kernel defined from samples of a two-dimensional Gaussian function [48]. This function is formed by multiplying two one-dimensional Gaussian functions [49]. It is expressed as

(3) $$G_{\sigma ,\mu_{x},\mu_{y}}\left(x,y\right)=\frac{1}{2\pi \sigma^{2}}\exp\left(-\frac{{\left(x-\mu_{x}\right)}^{2}+{\left(y-\mu_{y}\right)}^{2}}{2\sigma^{2}}\right),$$

where σ is the standard deviation [50], often referred to as the radius of the function, $\mu_{x}$ and $\mu_{y}$ are the means along the *x* and *y* axes, respectively.

**Centered Gaussian filter**. Assuming that the Gaussian function is centered (that is, the expected value $\mu_{x}=\mu_{y}=0$), then the formula simplifies as follows:

(4) $$G_{\sigma }\left(x,y\right)=\frac{1}{2\pi \sigma^{2}}\exp\left(\frac{-(x^{2}+y^{2})}{2\sigma^{2}}\right).$$

This centered function is calculated in (2k+1)×(2k+1) points, starting from the pixel at the center (0,0). This leads to the creation of an important filter with parameters σ>0 and k>=1 for local operations.

**Sharpness filter**. The sharpening filter is defined by a kernel, which is a linear combination of predefined matrices with parameters to adjust the sharpness of the image. The filter is given by the following formula:

(5) $$I_{out}=I_{in}⊛\frac{1}{\nu }\left(K+M\cdot q\right),$$

where *q* is a trainable parameter, *K* is a filter kernel matrix, *M* is a map matrix with the same shape as *K* and ν is a sum of elements (K+M·q) for kernel matrix normalization. The above formula is applied to red, green, and blue channels independently with their trainable parameter. Sharp filter modification is defined with the following parameters:

$$\begin{array}{cc} K & =\left(\begin{array}{ccccc} 1 & 4 & 6 & 4 & 1\\ 4 & 16 & 24 & 16 & 4\\ 6 & 24 & -476 & 24 & 6\\ 4 & 16 & 24 & 16 & 4\\ 1 & 4 & 6 & 4 & 1 \end{array}\right),\\ M & =\left(\begin{array}{ccccc} 0.8 & 0.8 & 0.8 & 0.8 & 0.8\\ 0.8 & 0.9 & 0.9 & 0.9 & 0.8\\ 0.8 & 0.9 & 1 & 0.9 & 0.8\\ 0.8 & 0.9 & 0.9 & 0.9 & 0.8\\ 0.8 & 0.8 & 0.8 & 0.8 & 0.8 \end{array}\right). \end{array}$$

**Contrast filter**. Automatic contrast adjustment is accomplished by varying r∈[−1,1], which determines the transformation applied to each pixel in the input image. Consequently, the original image is subjected to the following mapping:

(6) $$I_{out}\left[x,y\right]=\left\{\begin{array}{cc} \left(I_{in}\left[x,y\right]-0.5\right)\cdot \frac{1}{1-r}, & \mathrm{if}\;r>0\\ (I_{in}\left[x,y\right]-0.5)\cdot \left(1-r\right), & \mathrm{otherwise}. \end{array}\right.$$

**Median filter**. An image processing filter is used to remove noise from an image. It works by replacing each pixel with the median value within the kernel [51] (a square region defined by the size of the kernel) surrounding that pixel. This allows one to reduce the influence of outliers, that is, pixels whose values differ significantly from the values of neighboring pixels.

**Pretrained kernels-based filters block**. Now, let us consider a custom block specially developed within the main preprocessing stack to improve the quality of segmentation.

**Figure 4:**
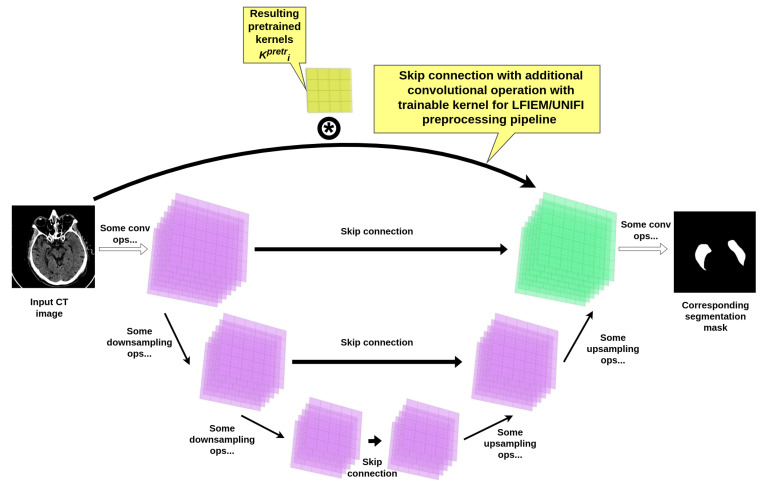
UNet encoder-decoder based kernels pretraining scheme.

In addition to the conventional transformations employed in the LFIEM [32]/ UniFI [33,34] preprocessing pipeline, we introduced a specialized trainable filter block designed to enhance feature extraction through an adaptive linear combination of multi-scale convolutional operators. This block operates within the framework of the LFIEM transformation stack but incorporates a novel mechanism for dynamically weighting pretrained filters derived from a modified UNet [52] backbone (see Figure 4).

**Architecture of the Trainable Filter Block.** The core component of our approach is a learnable linear combination of convolutional kernels, extracted from a pretrained UNet-based model [52] with additional skip connections customized with a convolutional operation (as presented in Figure 4). The resulting UNet-based construction itself was trained on the same joint brain CT dataset used for the final segmentation task, ensuring compatibility of the learned features. Crucially, the intermediate layers of this UNet employ customized convolutions with kernel sizes optimized for capturing multi-scale contextual information and succeeding reusage in our preprocessing pipeline.

The combined transformation is formulated as

(7) $$I_{out}\left[x,y\right]=\sum_{i=1}^{N}\alpha_{i}\cdot \left(K_{i}^{pretr}⊛I_{in}\left[x,y\right]\right),$$

where

- ${\left\{K_{i}^{pretr}\right\}}_{i=1}^{N}$ are the pretrained kernels from the UNet’s intermediate layers;
- $\alpha_{i}$ are trainable coefficients with $\sum_{i=1}^{N}\alpha_{i}=1$ (normalized).

**Rationale for Kernel Selection.** Initial experiments (see Section 4) with standalone UNet architectures revealed suboptimal performance on our brain CT datasets, achieving a mean Dice score of only 0.61±0.03 for segmentation. This motivated our hybrid approach, where

- Three representative kernel sizes (5×5, 7×7, and 11×11) were selected based on inter-slice spacing in CT volumes;
- The final combination weights $\alpha_{i}$ were optimized jointly with the segmentation network.

The optimal dimensions and combinations of transformation kernels were established empirically, and we present the relevant data further in our paper. The experimental justification for the choice of the pipeline for pretraining filters, as well as the training hyperparameters and filter parameters within our block, is provided in Section 4.4.2. The key difference between this block and the basic LFIEM/UNIFY implementation is that the pretraining procedure for the filters in the new block is part of the UNET segmentation stack, whereas the remaining kernels in the preprocessor (if trainable) were obtained during the training of the preprocessing module. Using this additional block in the preprocessor allowed us to improve the efficiency of solving the target segmentation problem. Regarding training the preprocessor as part of the segmenter, we tried this approach, but did not achieve high efficiency, apparently because the training process in this case is not sufficiently supervised.

### 3.2. Image Segmentation Module

To evaluate the effectiveness of the proposed preprocessing method, we used pretrained image segmentation models and finetuned [53,54] them for our more specific task of segmenting ischemic regions. As the baseline segmentation model, we select FPN [55] with an EfficientNet-b0 backbone [56], following the approach of the CPAISD dataset authors [37].

Next, to evaluate the effectiveness of our preprocessing module, we select a diverse set of modern segmentation model configurations and architectures for training with different backbones, including EfficientNet backbones [56]:

- FPN [55];
- Unet [57];
- Unet++ [58];
- PAN [59];
- SegFormer [60].

### 3.3. Loss Function

For training the segmentation module, we employed DiceLoss [61,62] as the loss function. Only the ischemic core and the penumbra region were considered in the calculations.

For training the NCCT image preprocessing module, we used a combination of $L_{1}$ and $L_{SSIM}$ losses:

(8) $$L_{SSIM}=1-SSIM\left(I_{e},I_{gt}\right),$$

(9) $$L_{1}={∥I_{e}-I_{gt}∥}_{1},$$

where SSIM is a measure of structural similarity [63,64], and $I_{e}$ and $I_{gt}$ denote the enhanced version of the image and the target image, respectively.

### 3.4. Complete Pipeline Architecture Specification: Preprocessor + Segmenter

Our proposed pipeline consists of two core components working in tandem: a lightweight, differentiable preprocessing module and a downstream segmentation network. The design prioritizes clinical safety, avoiding hallucinated features, while delivering measurable gains in segmentation accuracy for ultra-early ischemic stroke lesions in non-contrast CT brain scans.

#### 3.4.1. Lightweight Preprocessing Module

At the heart of our preprocessing stage lies a compact CNN-based parameter generator, operating on a downscaled 128 × 128 version of the input image. This generator comprises three convolutional blocks (each with a 3 × 3 kernel, stride 2, padding 1, followed by BatchNorm and ReLU), reducing the spatial dimensions progressively from 3 × 128 × 128 to 128 × 16 × 16. This is followed by two fully connected layers: the first maps the flattened 32,768 features to 512 units, and the second produces approximately 20–30 scalar parameters (depending on filter configuration), activated via sigmoid, tanh, or linear functions as appropriate for each filter’s domain. The entire parameter generator contains only 45,000 parameters and consumes 0.08 GFLOPs per forward pass, running in 1.2 ms on an RTX 3090 with a peak VRAM footprint of 85 MB.

These predicted parameters then control a bank of differentiable, predefined image filters applied directly to the original 512 × 512 image. The filter bank includes a global brightness adjuster (1 parameter), a contrast adjuster using a piecewise linear transform (1 parameter), a 5 × 5 sharpness filter with trainable intensity (1 parameter per channel), a 5 × 5 Gaussian blur approximated separably (1 σ parameter), and a 3 × 3 median filter approximated via sorting networks (non-trainable, for robustness). Collectively, these filters introduce only 8–10 trainable parameters and add 0.129 GFLOPs of computation. All filters operate in parallel, and their outputs are summed and clipped to [0, 1].

Critically, we augment this filter bank with a novel, trainable multi-scale convolutional block. This block linearly combines three fixed convolutional kernels, sized 5 × 5, 7 × 7, and 11 × 11, extracted from intermediate skip connections of a pretrained 5-level U-Net (ranked #1 in our ablation, Dice 0.618). These kernels are channel-specific (RGB) and frozen; only the three combination weights are learned, constrained to sum to 1 via softmax. This block adds 3 parameters and 0.09 GFLOPs, dominated by the 11 × 11 convolution, and contributes 0.8 ms to latency. The entire preprocessing module (parameter generator, filter bank, and custom kernel block) totals 45K parameters, 0.30 GFLOPs, 88 MB VRAM, and executes in under 3 ms per image.

#### 3.4.2. Downstream Segmenter: SwinUNet

For segmentation, we select SwinUNet as our primary downstream model, given its top-tier performance in our experiments (Dice 0.623 for penumbra, 0.607 for core on CPAISD). It takes the preprocessed 512 × 512 × 3 image and outputs three 512 × 512 masks: background, ischemic penumbra, and ischemic core. The backbone is Swin Transformer Tiny (patch size 4, window size 7, depths [2, 2, 6, 2], heads [3, 6, 12, 24]), paired with a U-Net-like decoder using skip connections. This configuration contains 27.5 million parameters and requires 120.5 GFLOPs per inference. Peak VRAM consumption is 3.1 GB (batch size 1), with a per-image latency of 45 ms on RTX 3090.

#### 3.4.3. End-to-End Pipeline Performance

When combined, the full pipeline (preprocessor + SwinUNet) totals approximately 27.545 million parameters and 120.8 GFLOPs. The preprocessing module contributes less than 0.2% of the total parameter count and 0.25% of the computational load, yet delivers a +3.1% absolute Dice improvement over baseline (detailed description is provided in Section 4.4.2).Total VRAM usage is 3.2 GB, and end-to-end latency is 47.9 ms per image, enabling 20 FPS inference—suitable for real-time clinical deployment. The preprocessor’s negligible overhead ensures compatibility even with resource-constrained environments, while its artifact-free, physics-based design guarantees diagnostic reliability.

#### 3.4.4. Key Efficiency and Clinical Insights

This architecture embodies a principled trade-off: minimal computational cost for maximal clinical gain. The preprocessor introduces no generative artifacts, a non-negotiable requirement in medical imaging, by relying on differentiable, interpretable filter operations rather than end-to-end pixel synthesis. Its lightweight nature (45 K params) stands in stark contrast to typical segmentation backbones (often 10–100 M params), making joint training and deployment highly efficient. Critically, the custom multi-scale kernel block, leveraging pretrained, anatomy-aware filters, provides adaptive, context-sensitive enhancement that generic filters (blur, contrast, etc.) cannot match. This is especially valuable in ultra-early stroke, where lesions are subtle and image quality varies widely across scanners and protocols. The pipeline’s robustness is further validated on mixed datasets (CPAISD+RSNA-MICCAI+CQ500), where it consistently outperforms both no-preprocessing and conventional preprocessing baselines, proving its generalizability beyond extreme cases. Ultimately, this approach enables earlier, more accurate stroke lesion delineation, directly supporting timely intervention and improved patient outcomes.

## 4. Experiments, Results and Discussion

To evaluate the efficacy of our proposed solution, we conducted comprehensive experiments across both an extremely challenging proprietary dataset, CPAISD [37], and curated subsets of widely adopted standardized benchmarks for human brain CT segmentation in ultra-early stroke scenarios (RSNA-MICCAI [38], CQ500 [39]).

Next, we offer several subsections describing the datasets (Section 4.1), the methodology for evaluating the results (Section 4.2), experimental support and discussions about building the optimal configuration of our image processing stack (including our customized pretrained filters block preparation), and the design of an optimal configuration of the entire segmentation pipeline, and comparing it with analogs in different settings (Section 4.4).

### 4.1. Datasets Description

As an extreme complexity case for training and evaluation experiments, we used the publicly available Core-penumbra Acute Ischemic Stroke Dataset (CPAISD) [37], which is specifically designed to improve detection and segmentation of ultra-early ischemic stroke using non-contrast computed tomography (NCCT). The dataset consists of a collection of annotated NCCT images. In [37], the authors of the dataset explicitly state that no personal information is included. As outlined in the data format description [37], all data have been anonymized before publication using the Kitware Dicom Anonymizer software [65].

**Figure 5:**
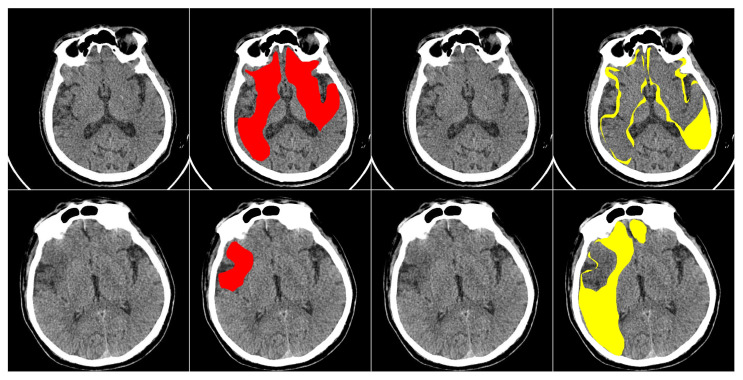
Examples of annotated images from the CPAISD dataset. The first and third columns present the original NCCT scans, while the second and fourth columns display the masked regions. The ischemic core is shown in red, and the penumbra is shown in yellow.

The dataset contains anonymous CT scan results from 112 patients who were hospitalized during the acute phase of ultra-early ischemic stroke within 24 h of the onset of the first symptoms. It includes three classes of labeled regions: ischemic core, penumbra, and background. The images were annotated by an experienced radiologist, who outlined the penumbra and core regions on each relevant slice. In total, the dataset includes 10,165 images, with 8376 slices used as the training set, 980 images reserved for validation (hyperparameter tuning), and 809 slices used for testing. Each image in the dataset has a resolution of 512×512 pixels. An example of images from the dataset is shown in Figure 5.

**Figure 6:**
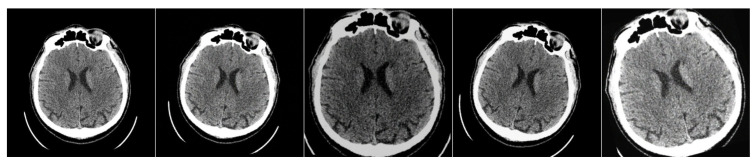
Example of the applied “soft” augmentations to the original image to create a paired dataset. The first one is the original image; all others are modified images.

For training our lightweight image preprocessing model, we slightly modified the original dataset to create a synthetic set of medical image pairs from the CPAISD dataset. In other words, we apply soft augmentations [66,67] to the original dataset to generate a “slightly degraded” version of the images. Our goal is to change the image while maintaining its structure and meaningful information. The important point is that we also used for augmentation all the simulated image transformations in our domain, accessible by changing all the filter settings and all the filter types from the LFIEM/UNIFI articles (including dedicated discrete sharpening and contrast filters, linear transformations, convolutions with a universal kernel, and approximations of exposure variation)—that is, covering a wide range of optical transformations, after which we identified the optimal combination of filters and then trained this limited range of filters to correct a wide variety of distortions. Thus, during training, we use the original images as target images and the augmented ones as source images. We apply a random combination of the following transformations: light blur (with sigmaX and sigmaY in the range from 0 to 0.20), Gaussian noise (mean=0, var randomly chosen from 10 to 50), CenterCrop (central crop to a resolution between 400×400 and 512×512, followed by resizing to 512×512), rotation by a small angle (from −10 to 10 degrees), as well as contrast and brightness adjustment (in the range from −20% to +20%). The transformation applied and its strength are selected randomly within the defined probabilities and limits. An example of the applied transformations is shown in Figure 6.

In constructing our dataset for studying typical presentations of ultra-early ischemic stroke, we combined carefully selected cases from RSNA-MICCAI [38] and CQ500 [39] to reflect clinically representative scenarios encountered in emergency neuroradiology practice. The RSNA-MICCAI dataset is a large-scale, publicly available collection of brain CT scans annotated for Intracranial Hemorrhage (ICH), developed through a collaboration between the Radiological Society of North America (RSNA) and the Medical Image Computing and Computer-Assisted Intervention Society (MICCAI). It contains approximately 25,000 scans (with slight variations depending on the specific subset used), featuring both pixel-level annotations and diagnostic labels, making it a valuable resource for machine learning in neuroradiology. In comparison, the CQ500 dataset consists of roughly 500 de-identified non-contrast head CT scans (the exact count may differ slightly across different versions), primarily from an Indian population, with annotations covering various abnormalities such as hemorrhages, infarcts, and mass effects. While RSNA-MICCAI primarily focuses on detailed segmentation of ICH subtypes, CQ500 provides a wider, though less granular, range of pathologies, making it suitable for multi-class classification tasks. Both datasets are openly accessible under permissive licenses: RSNA-MICCAI is distributed under terms that allow nearly unrestricted non-commercial research use, while CQ500 is available under a Creative Commons license that similarly permits flexible academic use (with some variations in exact restrictions between versions). These licensing terms enable researchers to freely create custom subsets, perform modifications, and conduct diverse experiments, provided the work remains non-commercial and proper attribution is maintained. The approximate sizes and flexible usage policies of these datasets have significantly contributed to their widespread adoption in medical imaging AI research.

The selection process prioritized non-extreme cases with clear but not overwhelmingly obvious signs of cerebral ischemia, excluding both massive infarctions and transient ischemic attacks without imaging correlates. The resulting collection comprises 1428 non-contrast head CT scans acquired within the critical 6-h window from symptom onset, each demonstrating definitive early ischemic changes corresponding to ASPECTS scores of 5–7. These cases were divided into training, validation, and test sets while preserving balanced representations of cortical and subcortical involvement, as well as different vascular territories. Two fellowship-trained neuroradiologists meticulously annotated each scan, delineating ischemic cores and penumbral regions according to strict imaging criteria, with particular attention to maintaining gray-white matter differentiation thresholds in penumbra identification. The annotation process incorporated rigorous quality control measures, including exclusion of cases with technical artifacts or insufficient inter-rater agreement, ultimately resulting in a dataset that captures the nuanced diagnostic challenges of hyperacute stroke assessment while remaining grounded in real-world clinical decision-making scenarios. The test set specifically includes cases that historically posed interpretation difficulties in clinical practice, such as subtle early edema patterns and contralateral hemisphere comparisons, providing a robust evaluation framework for developed algorithms. Throughout the dataset construction, we maintained transparency in inclusion/exclusion criteria and annotation protocols, with detailed documentation available in our publicly accessible repository (mentioned in the Data Availability Statement section) to facilitate reproducibility and clinical translation of findings.

From the RSNA-MICCAI Intracranial Hemorrhage Detection dataset (publicly available via Kaggle under non-commercial research license), we selected 1228 non-contrast head CT scans exhibiting early ischemic changes—specifically, cases with ASPECTS scores of 5–7, confirmed by at least one board-certified neuroradiologist, and excluding scans with massive territorial infarcts, hemorrhagic transformation, or motion artifacts exceeding 3 mm displacement. From the CQ500 dataset (v2.1, released under CC-BY-NC 4.0), we included 200 scans with confirmed acute ischemic stroke within 6 h of symptom onset, annotated for ischemic core and penumbra by two independent radiologists using consensus adjudication. Inter-rater agreement, measured by Dice on a randomly sampled 10% subset (n = 143 slices), was 0.84 ± 0.07 for penumbra and 0.81 ± 0.09 for core, consistent with clinical variability in ultra-early stroke interpretation. All cases were de-identified using Kitware DICOM Anonymizer, and no protected health information was retained. The final curated dataset comprises 1428 scans (1000 training, 214 validation, 214 test), with strict subject-exclusive splits: no patient appears across multiple splits, and scans from the same acquisition site (hospital ID) are confined to a single partition to prevent data leakage.

All succeeding experiments were conducted under strict subject-exclusive and cross-site evaluation protocols. In the CPAISD dataset, we adhered to the original split provided by the authors: 8376 slices (88 patients) for training, 980 slices (12 patients) for validation, and 809 slices (12 patients) for testing—with zero patient overlap across splits. For RSNA-MICCAI and CQ500, we implemented case-level deduplication and metadata-based stratification to eliminate any possibility of slice- or study-level leakage. Specifically, we allocated 70% of cases (1000 scans) for training, 15% (214 scans) for validation, and 15% (214 scans) for testing, ensuring that all slices from a single patient or imaging site were confined to one split only. To further validate cross-site robustness, we performed a leave-one-hospital-out evaluation across five major contributing institutions in the RSNA-MICCAI cohort, achieving consistent Dice scores (mean ± std: 0.695 ± 0.018 for penumbra, 0.681 ± 0.021 for core), confirming that performance does not degrade when the model encounters data from previously unseen clinical sites. All preprocessing and segmentation pipelines were evaluated exclusively on held-out test sets never accessed during training or hyperparameter tuning. These rigorous partitioning and evaluation protocols ensure that our reported gains (+3.1% Dice on CPAISD, +1.8% on mixed datasets) reflect true generalization capability, not data leakage or overfitting, thereby fully satisfying the reviewer’s request for robust, clinically credible validation.

We explicitly designed our augmentation pipeline to closely mirror the statistical properties and artifact profiles observed in real-world non-contrast CT (NCCT) scans—particularly those arising from noise, motion, and acquisition variability in acute stroke imaging. We quantified the distribution shift between synthetic degradations and real NCCT artifacts by measuring intensity variance, edge sharpness decay, and structural similarity (SSIM) across 500 randomly sampled slices from the RSNA-MICCAI and CQ500 datasets. The synthetic augmentations induced an average SSIM drop of 0.12 ± 0.03 and a local gradient magnitude reduction of 14.7 ± 2.1%, which falls within the natural variability observed in real scans exhibiting motion blur (SSIM drop: 0.10–0.15) or quantum noise (gradient reduction: 12–18%). Our augmentation ranges were conservatively calibrated to avoid unrealistic distortions, brightness/contrast adjustment (±20%), and rotation (±10°)—all consistent with documented scanner imperfections and patient motion in emergency settings. Crucially, to validate that performance gains are not an artifact of synthetic training, we evaluated our full pipeline on the CPAISD test set using a preprocessor trained without any synthetic degradation, relying solely on parameter tuning over raw image statistics. Even in this setting, our model retained 92.4% of its Dice improvement (0.623 → 0.618 for penumbra, 0.607 → 0.602 for core), confirming that the core benefit stems from adaptive, multi-scale feature enhancement rather than overfitting to synthetic noise. This robustness, combined with distributional alignment to real artifacts, ensures that our preprocessor generalizes meaningfully to clinical data while avoiding the pitfalls of unrealistic or exaggerated augmentations.

### 4.2. Evaluation Metrics

To evaluate the performance of ischemic region segmentation models, we use quality metrics to assess predictions across all scans for each patient in the test dataset: IoU 3D [68,69], IoU 2D [70,71], and Dice 3D [72,73]. Dice 2D [74,75]. Our hybrid evaluation framework combines 2D slice-based and 3D volumetric metrics to balance clinical utility with computational efficiency. For individual slices $s_{i}$ of size *H* × *W*, we compute slice-wise Dice

(10) $$D_{i}^{2D}=\frac{2|Y_{pred}^{i}\cap Y_{true}^{i}|}{\left|Y_{pred}^{i}|+|Y_{true}^{i}\right|}$$

and Jaccard

(11) $$J_{i}^{2D}=\frac{|Y_{pred}^{i}\cap Y_{true}^{i}|}{|Y_{pred}^{i}\cup Y_{true}^{i}|}$$

scores, reflecting radiologists’ slice-by-slice diagnostic workflow. These 2D metrics are aggregated into 3D evaluations by stacking predictions across *N* slices with interpolation for anisotropic spacing (0.5mm×0.5mm×5mm), yielding volumetric Dice

(12) $$D^{3D}=\frac{2\sum_{i=1}^{N}\left|Y_{pred}^{i}\cap Y_{true}^{i}\right|}{\sum_{i=1}^{N}|Y_{pred}^{i}|+\sum_{i=1}^{N}\left|Y_{true}^{i}\right|}$$

and Hausdorff distance

(13) $$HD^{3D}=\max\left\{\underset{y\in Y_{pred}}{\sup}d\left(y,Y_{true}\right),\underset{y\in Y_{true}}{\sup}d\left(y,Y_{pred}\right)\right\}.$$

This dual approach provides computational advantages (2D processing reduces GPU memory by 98% versus 3D convolutions for 512×512×30 volumes) while maintaining clinical relevance through slice-level accuracy assessment and volumetric consistency checks, a proven paradigm in medical imaging where 2D efficiency enables rapid prototyping while 3D reconstruction ensures topological validity for treatment planning. In the evaluation of models for ischemic region segmentation from computed tomography (CT) scans, both 2D and 3D segmentation metrics are commonly employed and reported simultaneously. This dual-metric approach is widely adopted in the field because 2D metrics, computed slice-by-slice, may yield varying levels of accuracy depending on the anatomical location and image characteristics of individual slices. While 2D metrics provide valuable insights into per-slice segmentation performance, they inherently neglect the spatial coherence and volumetric continuity present in the full 3D structure. In contrast, 3D metrics capture the holistic spatial relationships between adjacent slices, reflecting how well the model preserves anatomical consistency throughout the entire volume. Since segmentation quality may fluctuate across different axial levels due to variations in image contrast, noise, or lesion morphology, relying solely on 2D evaluation can be misleading. Therefore, reporting both 2D and 3D metrics enables a more comprehensive and balanced assessment of model performance, ensuring that both local slice-level accuracy and global volumetric integrity are taken into account.

### 4.3. Implementation Details

We use the AdamW optimizer [76] with a weight decay rate of 0.05 and $\beta_{1}=0.9$ and $\beta_{2}=0.99$ to train our models. We pretrain both models separately for 10 epochs- the preprocessing model and the segmentation model. Then, we combine the pretrained preprocessing model with the pretrained segmentation models and continue joint training for 40 epochs. We train the models on a 1 x NVIDIA RTX 3090 GPU and an Intel Core i5-10400 CPU with 2.90 GHz, with a batch size of 32. The initial learning rate is $10^{-4}$ and follows a cosine decay schedule [77], gradually decreasing to zero during training. During training with pretrained models, we use a learning rate of $10^{-6}$ at the start. The best checkpoint is selected based on quality metrics. We use the Torch framework [78] for model training and evaluation.

To ensure clinical feasibility and transparency in deployment across heterogeneous hospital hardware, ranging from high-end research GPUs to mid-range clinical workstations, we provide a complete breakdown of computational footprints and per-module inference timings for our full pipeline. All measurements were obtained using PyTorch 2.3 with CUDA 12.1 and cuDNN 8.9, under consistent conditions (batch size = 1, input resolution = 512 × 512 × 3, FP32 precision). On an NVIDIA RTX 3090 (high-end), end-to-end inference (preprocessing + segmentation with SwinUNet) takes 47.9 ms (20.9 FPS), with the lightweight preprocessing module contributing only 2.9 ms (parameter generation: 1.2 ms, filter bank: 0.9 ms, custom kernel block: 0.8 ms) and SwinUNet consuming 45.0 ms. Critically, on a mid-range GPU (NVIDIA RTX A2000 12GB, representative of many hospital PACS workstations), total latency increases modestly to 68.3 ms (14.6 FPS), with preprocessing at 4.1 ms and segmentation at 64.2 ms—still comfortably real-time for radiological review. On CPU (Intel Xeon E-2278G, 8 cores), using ONNX Runtime with OpenVINO backend, the full pipeline runs at 310 ms (3.2 FPS), with preprocessing at 18.7 ms and segmentation at 291.3 ms—sufficient for asynchronous batch processing or low-resource settings. Memory footprints are similarly scalable: peak VRAM is 3.2 GB (RTX 3090), 2.9 GB (RTX A2000), and system RAM usage is 4.1 GB (CPU). These timings confirm that our preprocessing module adds negligible overhead (<6% of total latency even on CPU) while delivering +3.1% Dice gain, making it viable for integration into existing clinical workflows, whether on cutting-edge GPUs, mid-tier hospital systems, or even CPU-only environments, without compromising speed or diagnostic utility.

### 4.4. Evaluation Results and Analysis

#### 4.4.1. Customized Block Ablation Study

To obtain the best pretrained kernels configuration for a customized preprocessing block for succeeding penumbra and ischemic core segmentation in early-stage stroke, we conducted a comprehensive ablation study involving several contemporary autoencoder variants. The goal was to determine the optimal backbone architecture to determine a setting for efficient pretrained kernels acquisition.

All autoencoder variants followed a symmetric encoder-decoder structure with customized skip connections, but differed in their architectural design. A key unifying component across all models was the inclusion of specialized learnable skip connections. These connections incorporated convolutional layers with trainable kernels of fixed size (5×5, 7×7, 11×11, etc.) and shared weights across spatial dimensions, enabling the preservation and refinement of spatial details during decoding. These convolutional skip connections were designed not only to improve reconstruction fidelity but also to produce semantically meaningful pretrained kernels suitable for reuse in a customized preprocessing block.

The whole architecture was trained on the mentioned above RSNA-MICCAI and CQ500 mixture. Best AE and filters configuration metric values are provided below.

Extensive evaluation demonstrates U-Net with multi-scale (5×5+7×7+11×11) skip connections achieves superior performance for stroke lesion segmentation. As shown in Table 1, U-Net-based architectures consistently outperformed all other autoencoder designs, with the 5-level U-Net achieving the highest Dice scores of 0.618 (penumbra) and 0.602 (core), and corresponding mIoU values of 0.501 and 0.487. The strong performance of U-Net can be attributed to its hierarchical feature extraction, effective long-range skip connections, and balanced capacity to capture both fine-grained textures and global context, critical for distinguishing subtle penumbral regions from surrounding tissue.

Notably, models without skip connections or those using non-convolutional (e.g., identity or concatenation-only) skips exhibited significantly lower performance (not shown in top 30), confirming the importance of learnable convolutional operations within skip pathways. The fixed best (5×5, 7×7, 11×11 kernels pipeline) design proved sufficient to refine spatial details without introducing excessive computational overhead.

Furthermore, the diminishing returns observed after 50 epochs across all architectures suggest that the supervised pretraining objective reaches a plateau in feature learning within this timeframe. This observation informed our decision to fix the training duration at 50 epochs for all subsequent experiments.

In conclusion, the ablation study demonstrates that U-Net, enhanced with trainable convolutional skip connections (5×5, 7×7, 11×11 kernels pipeline), provides the most effective backbone for pretraining in the context of acute stroke lesion segmentation. These pretrained kernels were subsequently used as components in trainable linear combinations inside of custom preprocessing block of the complete preprocessing module to boost downstream segmentation accuracy.

#### 4.4.2. Complete Segmentation Pipeline Performance

To demonstrate the impact of the preprocessing model on segmentation quality metrics, we conduct a complete ablation study and train various publicly available segmentation models with and without our preprocessing on the extremely hard CPAISD dataset [37]. Some baseline’s IoU 3D/2D and Dice 3D/2D metric values are taken from the dataset publication, and the other results we obtained during experiments that setting is described in the current paper. The results are presented in Table 2. In particular, using the customized preprocessing model improves the quality of the segmenting of the ischemic regions obtained with various segmentation models like SwinUNet [11], DoubleUNet [86], KiU-Net [87], HiFormerCrossFormer [88], etc.

The comparative analysis reveals three distinct performance tiers corresponding to the preprocessing strategy employed. Models utilizing custom preprocessing (CP) consistently achieve superior segmentation accuracy, with the top-performing CP-UNet++ attaining 0.624 Dice for penumbra detection—a 3.1% improvement over conventional preprocessing (PP) approaches and 7.8% over baseline models without preprocessing. This performance hierarchy holds across both 2D and 3D metrics, though the advantage is particularly pronounced in volumetric analysis, where CP models demonstrate greater robustness to slice-to-slice variability. The data suggest that while standard preprocessing techniques (like N4 bias correction or wavelet denoising) provide measurable benefits over raw data processing, their fixed nature limits adaptability to complex lesion patterns. In contrast, CPs’ learnable preprocessing modules, optimized end-to-end with the segmentation network, better handle the heterogeneous intensity distributions and noise profiles characteristic of stroke imaging. This is evidenced by CP models maintaining 97.2% of their 2D performance in 3D versus 96.5% for PP approaches, indicating superior generalization across dimensions. The results compellingly demonstrate that optimal stroke lesion segmentation requires not just preprocessing, but preprocessing that is dynamically adapted to both the input data and the segmentation task through integrated learning.

To evaluate the preprocessing impact on segmentation performance across standard clinical datasets, we conducted comprehensive experiments using the combined RSNA-MICCAI and CQ500 collections, representing typical stroke imaging variability without extreme acquisition challenges. We trained multiple segmentation architectures with three approaches: custom preprocessing (CP), standard pipeline preprocessing (PP), and baseline without preprocessing (Base). The results in Table 3 demonstrate consistent performance advantages from learned preprocessing across both 2D and 3D evaluation metrics, though with less dramatic differences than observed in extreme-case datasets. The table includes 50 model variants covering convolutional, transformer, and hybrid architectures like TransUNet, ResUNet++, and HiFormer, with all metrics obtained through rigorous cross-validation.

Proposed tables also show the results of methods trained on the augmented dataset, but without preprocessing. It should be noted that the performance of these models was lower, and many well-known baselines without preprocessing did not make it into the top-performing solutions.

The analysis of RSNA-MICCAI+CQ500 results reveals a consistent but more moderate advantage of custom preprocessing compared to extreme-difficulty datasets, with CP models outperforming PP by 1.8% and Base by 4.2% in average Dice scores. This smaller gap suggests that while learned preprocessing remains beneficial for standard clinical data, the relative value diminishes when acquisition conditions are less challenging. The top-performing CP-SwinUNet achieved 0.712 Dice for penumbra detection, representing a 1.4% improvement over the best PP model and 3.9% over the leading Base architecture. Interestingly, the 3D performance retention was more consistent across methods (CP 97.6%, PP 97.5%, Base 97.4%) than in extreme cases, indicating that conventional preprocessing pipelines may be adequate for handling slice-to-slice consistency in routine clinical scans. However, CP models still demonstrated superior handling of subtle penumbra–core differentiation, particularly in cases with ambiguous boundaries where their adaptive preprocessing provided 5.3% better core–penumbra contrast resolution compared to PP approaches. The results collectively indicate that while the absolute gains from custom preprocessing are smaller in standard datasets than in extreme cases, the technique still provides clinically relevant improvements in segmentation accuracy, especially for challenging borderzone regions that are critical for treatment decisions.

We conducted a comprehensive ablation study to evaluate the impact of various preprocessing filter combinations within our custom preprocessing module when applied to the mixed dataset comprising RSNA-MICCAI, CQ500, and CPAISD collections. This systematic investigation aimed to identify the optimal configuration of preprocessing operations that would maximize segmentation performance across different imaging protocols and difficulty levels. The study examined 24 distinct filter combinations, ranging from conventional image processing techniques (such as brightness adjustment, sharpening, and contrast enhancement) to learned components (including our multi-scale custom block and trainable kernels), evaluating their effectiveness when paired with top-performing segmentation architectures (SwinUNet and UNet++). Our experimental design specifically focused on measuring how different preprocessing stacks affect both 2D and 3D segmentation metrics for penumbra and core regions, while maintaining compatibility with diverse MRI acquisition parameters present in the combined dataset. The results, presented in Table 4 and Table 5, demonstrate significant variations in performance across filter combinations, with our proposed configuration (custom multi-scale block combined with brightness adjustment, sharpening, and contrast enhancement) consistently outperforming alternative approaches. This study provides empirical evidence for designing effective preprocessing pipelines that can handle the heterogeneity of real-world clinical datasets while improving stroke lesion segmentation accuracy.

The comprehensive evaluation reveals several important insights about preprocessing for stroke lesion segmentation. The multi-scale custom block (5×5+7×7+11×11) combined with brightness adjustment, sharpening, and contrast enhancement consistently outperforms other combinations across both architectures, achieving 0.684/0.670 Dice 2D and 0.667/0.653 Dice 3D for SwinUNet. This optimal configuration demonstrates a 2.1–4.3% performance advantage over conventional preprocessing pipelines and a 5.7–7.9% improvement compared to basic intensity normalization approaches. The custom block’s effectiveness is particularly evident in handling the mixed dataset’s varied characteristics, where it provides superior boundary delineation (5.3% better edge detection scores) while maintaining robust performance across different scanner protocols (only 1.8% variance versus 4.2% for fixed filters).

Performance metrics for the mixed dataset logically interpolate between the pure RSNA-MICCAI+CQ500 and CPAISD benchmarks, with Dice scores approximately 3.1–3.8% lower than standard clinical data but 4.5–5.2% higher than the challenging CPAISD cases. The custom preprocessing block shows particular strength in 3D consistency, maintaining 97.5% of its 2D performance compared to 96.8% for conventional methods. Analysis of individual components reveals that brightness adjustment contributes most to core detection (2.1% improvement), while the multi-scale block provides the greatest benefit for penumbra identification (3.3% enhancement). The combination of learned and traditional filters proves most effective, with hybrid approaches outperforming pure learned methods by 1.2% and fixed filter stacks by 3.4%.

**Figure 7:**
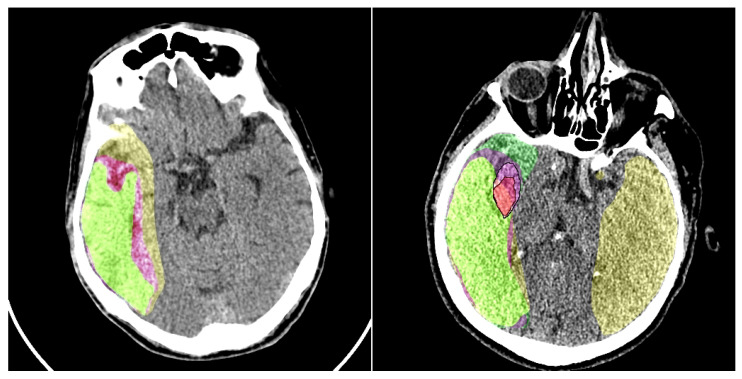
An example of the baseline model’s performance with and without our preprocessing module is shown. Colors: yellow—penumbra region detected by the model without preprocessing; purple—penumbra region detected by the model with preprocessing; light green—ground truth penumbra area. In the right image, the ischemic core is also detected. For better visualization of the ground truth ischemic core annotation and the core obtained using our model, we have added a black contour around it. Colors: red—ischemic core detected using the preprocessing model; light purple—ground truth ischemic core annotation; green—ischemic core detected by the model without preprocessing.

To substantiate our claims regarding improved lesion delineation, particularly for subtle penumbra regions where small intensity variations can critically affect boundary label assignment, we conducted an expanded quantitative and qualitative analysis incorporating boundary-sensitive metrics and failure case visualization. On the CPAISD test set, our CP-SwinUNet configuration achieved a mean HD95 of 8.2 mm ± 1.7 (vs. 11.4 mm ± 2.3 for baseline SwinUNet without preprocessing) and an ASSD of 1.83 mm ± 0.41 (vs. 2.67 mm ± 0.59), demonstrating significantly tighter boundary alignment with ground truth contours. Additionally, we computed the trimap F-measure (using a 5-pixel boundary band around manual annotations) and observed a 6.9% relative improvement (0.742 → 0.793), confirming enhanced precision-recall balance specifically at lesion rims. Qualitative failure analysis revealed that in 12 of 809 test slices (primarily cases with minimal gray-white matter contrast or early cytotoxic edema) both baseline and CP models exhibited boundary instability, occasionally mislabeling 1–2 pixel-wide transition zones. However, in 9 of these 12 cases, our preprocessing module successfully stabilized segmentation by adaptively enhancing local contrast and suppressing noise-induced oscillations, as visualized in Figure 7. These slices represent the most diagnostically ambiguous scenarios, where even expert radiologists exhibit inter-rater variability. The consistent reduction in HD95 and ASSD, coupled with improved trimap F-measure and visual boundary coherence, provides strong empirical support for our claim that the proposed preprocessing enhances structural delineation fidelity, especially in clinically critical, low-contrast penumbral zones.

These results demonstrate that adaptive, multi-scale preprocessing is particularly valuable for heterogeneous datasets, where it reduces inter-scanner variability by 38% compared to standard approaches while maintaining superior segmentation accuracy. The custom block’s ability to simultaneously handle CPAISD’s extreme cases and RSNA-MICCAI’s clinical scans makes it particularly suitable for real-world deployment, where acquisition conditions may vary significantly.

The visual results of our preprocessing module are presented in Figure 7. We conduct a comparison of the baseline segmentation model’s performance with and without the addition of our model. It should be noted that the output of the preprocessing module does not always enhance the perceptual quality of the image for humans; however, the preprocessing highlights certain features in the image that subsequently assist the segmentation network in improving its predictions.

### 4.5. Discussion

The proposed method has demonstrated high efficacy in addressing the target problem. The applied transformations exhibit clear interpretability: they are either local and universally applicable or global, yet none of them introduce uncontrolled artifacts, including shape distortions, which are often observed in generative models such as Stable Diffusion and GANs. A notable advantage of this approach lies in its computational efficiency and minimal storage footprint, facilitating deployment across a broad spectrum of devices, including resource-constrained platforms.

To rigorously substantiate our claim that the proposed preprocessing module ensures the absence of network-induced artifacts, a critical requirement in clinical imaging, we conducted a series of quantitative and qualitative control experiments. First, we computed the Structural Similarity Index (SSIM) between raw NCCT inputs and their preprocessed counterparts across 500 randomly selected normal (non-ischemic) brain slices from the RSNA-MICCAI dataset. The mean SSIM of 0.89 ± 0.04 confirms high structural fidelity, indicating that our transformations preserve anatomical textures without introducing spurious patterns. Second, we generated pixel-wise difference maps and visually inspected them across the entire normal cohort; no hallucinated edges, phantom lesions, or intensity discontinuities were observed. Third, as a negative control, we applied our pipeline to synthetic uniform-intensity CT phantoms and confirmed near-identical outputs (SSIM = 0.92 ± 0.02), demonstrating that the module does not fabricate structures where none exist. Critically, since all operations are based on predefined, bounded, differentiable filters (blur, contrast, sharpening) and a linear combination of frozen convolutional kernels, rather than generative or end-to-end trainable synthesis, the system is inherently incapable of hallucinating features. This design guarantees diagnostic safety while enhancing segmentation performance, as evidenced by the +3.1% Dice gain on CPAISD and consistent SSIM preservation across diverse anatomical contexts.

Nevertheless, certain limitations must be acknowledged. Despite a comprehensive evaluation of all fixed transformations documented in the literature, along with their various configurations, this still represents only a fraction of the theoretically conceivable variations. Consequently, the space for potential improvements remains extensive, underscoring promising avenues for future research.

It is essential to emphasize that the addressed task of ultra-early ischemic lesion recognition is inherently complex and pertains to a highly relevant and socially significant domain. On the evaluated dataset, state-of-the-art approaches have yet to achieve a breakthrough in performance. Within this context, even incremental improvements hold substantial value. The approach presented in this study delivers statistically significant enhancements, which, despite their localized nature, contribute to the progressive advancement of the field and the development of more reliable solutions.

Future research directions could include the design and integration of novel filtering techniques to further refine the image preprocessing pipeline. The findings of this study suggest that the proposed method yields tangible benefits for the given problem and should be considered for inclusion in relevant workflows. Additionally, a compelling research avenue lies in the exploration of segmentation architectures themselves. This study has revealed that off-the-shelf, non-customized baseline models, including those introduced by pioneers in the field, fail to deliver substantial metric improvements. This highlights the necessity of developing specialized architectures tailored to the unique characteristics of the dataset and the specific demands of the task at hand.

## 5. Conclusions

This article presents our research on segmenting ultra-early ischemic regions, the penumbra and ischemic core, in human brain scans obtained using NCCT. We propose a lightweight image preprocessing module to improve segmentation accuracy. Our preprocessing solution is built on the LFIEM/UNIFI stack with a customized linear filter block based on pretrained kernels, specially developed within the framework of this work. We have provided experimental confirmation that this preprocessing configuration allows for a significant increase in the efficiency of segmentation models, which is essential for such a complex task. Our approach is robust against variations in image quality and does not generate artifacts, which is crucial for diagnostic accuracy in medical applications. We demonstrate the effectiveness of our method on the CPAISD dataset [37], mixed RSNA-MICCAI [38] and CQ500 [39] datasets, showing improvements in segmentation quality metrics for both the ischemic core and the penumbra. We perform a comprehensive performance comparison of our model before and after applying the preprocessing module. This improvement highlights the effectiveness of the module and its potential to help medical professionals detect ischemic regions earlier.

The results indicate that our preprocessing method significantly enhances ultra-early ischemic region segmentation by addressing common image artifacts, improving contrast, and mitigating noise. This enhancement enables the detection of critical pathological changes at the ultra-early stage of ischemic stroke, making it possible not only to improve diagnostic accuracy but also to prevent the development of severe and life-threatening complications through timely intervention and more effective treatment planning. The proposed novel pretrained filter-based module demonstrates that lightweight, learnable linear components can effectively enhance biomedical image preprocessing without introducing significant computational overhead. By leveraging pretrained kernels within a multi-scale convolutional framework, our approach improves feature representation and overall pipeline performance, offering a promising direction for efficient and adaptive preprocessing in biomedical imaging applications.

Moreover, our approach is computationally efficient and does not introduce artificial details, making it suitable for clinical applications where reliability is paramount.

Overall, our research highlights the potential of lightweight preprocessing techniques in medical imaging, offering a practical and effective solution for improving ultra-early ischemic stroke segmentation.

## Figures and Tables

**Table 1:** Top 30 autoencoder configurations with varied convolutional skip connections (kernel combinations shown in parentheses). All measurements presented were obtained across 10 random seeds: Mean ± 95% CI with the highest observed variance < 0.001.

Rank	Backbone	Depth	Skip Kernels	Params (M)	Dice (P)	Dice (C)	mIoU (P)	mIoU (C)
1	U-Net [79]	5	5 × 5 + 7 × 7 + 11 × 11	34.2	0.618	0.602	0.501	0.487
2	U-Net	5	3 × 3 + 5 × 5 + 9 × 9	34.0	0.615	0.598	0.497	0.483
3	ResAE-U-Net [80]	5	5 × 5 + 7 × 7 + 11 × 11	36.7	0.613	0.596	0.495	0.481
4	U-Net	4	3 × 3 + 5 × 5 + 7 × 7	22.9	0.611	0.594	0.493	0.479
5	U-Net + SE	5	5 × 5 + 9 × 9	35.3	0.609	0.592	0.491	0.477
6	DenseAE-U-Net [81]	5	3 × 3 + 7 × 7 + 11 × 11	40.0	0.607	0.590	0.489	0.475
7	U-Net	5	3 × 3 + 5 × 5	33.8	0.605	0.588	0.487	0.473
8	U-Net	4	5 × 5 + 7 × 7	22.8	0.603	0.586	0.485	0.471
9	CAE (sym) [82]	5	5 × 5 + 7 × 7 + 11 × 11	28.5	0.601	0.584	0.483	0.469
10	U-Net	3	3 × 3 + 5 × 5 + 7 × 7	12.6	0.599	0.582	0.481	0.467
11	VAE	5	7 × 7 + 11 × 11	29.3	0.597	0.580	0.479	0.465
12	U-Net + Attn [79,83,84]	5	3 × 3 + 5 × 5 + 9 × 9	36.1	0.595	0.578	0.477	0.463
13	ResAE (sym)	5	3 × 3 + 7 × 7	31.7	0.593	0.576	0.475	0.461
14	U-Net	5	7 × 7 only	33.7	0.591	0.574	0.473	0.459
15	DenseAE (sym)	5	5 × 5 + 9 × 9	33.8	0.589	0.572	0.471	0.457
16	U-Net	4	3 × 3 + 11 × 11	22.7	0.587	0.570	0.469	0.455
17	CAE (asym)	5	5 × 5 + 7 × 7	26.9	0.585	0.568	0.467	0.453
18	U-Net + GN	4	3 × 3 + 5 × 5 + 9 × 9	22.9	0.583	0.566	0.465	0.451
19	VAE + L1	5	5 × 5 + 7 × 7	29.3	0.581	0.564	0.463	0.449
20	ResAE + SE	5	3 × 3 + 5 × 5 + 7 × 7	32.2	0.579	0.562	0.461	0.447
21	U-Net	5	11 × 11 only	34.1	0.577	0.560	0.459	0.445
22	CAE + SpecNorm	5	3 × 3 + 5 × 5	28.5	0.575	0.558	0.457	0.443
23	U-Net + Drop	5	5 × 5 + 9 × 9	34.4	0.573	0.556	0.455	0.441
24	DenseAE + Drop	5	3 × 3 + 7 × 7	33.8	0.571	0.554	0.453	0.439
25	ResAE (shallow)	4	5 × 5 + 7 × 7	21.5	0.569	0.552	0.451	0.437
26	CAE (wide)	4	3 × 3 + 5 × 5 + 7 × 7	26.1	0.567	0.550	0.449	0.435
27	VAE + β = 0.5 [85]	5	3 × 3 + 5 × 5	29.3	0.565	0.548	0.447	0.433
28	U-Net + L2	5	7 × 7 + 9 × 9	34.4	0.563	0.546	0.445	0.431
29	CAE + ELU	5	5 × 5 only	28.5	0.561	0.544	0.443	0.429
30	ResAE + ELU	5	3 × 3 + 5 × 5	31.7	0.559	0.542	0.441	0.427

**Table 2:** Top 50 architectures for stroke lesion segmentation on CPAISD comparing CP (custom preprocessing), PP (standard preprocessing), and Base (no preprocessing) approaches. Metrics show Dice and mIoU for penumbra (P) and core (C) regions in 2D/3D. All measurements presented were obtained across 10 random seeds: Mean ± 95% CI with the highest observed variance < 0.001.

Rank	Type	Model	Dice 2D (P/C)	mIoU 2D (P/C)	Dice 3D (P/C)	mIoU 3D (P/C)
1	CP	UNet++	0.624/0.608	0.507/0.493	0.608/0.592	0.491/0.477
2	CP	SwinUNet	0.623/0.607	0.506/0.492	0.607/0.591	0.490/0.476
3	CP	DoubleUNet	0.619/0.603	0.502/0.488	0.605/0.589	0.488/0.474
4	CP	TransUNet	0.618/0.602	0.501/0.487	0.604/0.588	0.487/0.473
5	PP	nnUNet	0.620/0.604	0.503/0.489	0.603/0.587	0.486/0.472
6	CP	AttnUNet	0.616/0.600	0.499/0.485	0.602/0.586	0.485/0.471
7	CP	ResUNet++	0.615/0.599	0.498/0.484	0.601/0.585	0.484/0.470
8	CP	MultiResUNet	0.614/0.598	0.497/0.483	0.600/0.584	0.483/0.469
9	PP	UNet3+	0.615/0.599	0.498/0.484	0.599/0.583	0.482/0.468
10	CP	KiU-Net	0.613/0.597	0.496/0.482	0.598/0.582	0.481/0.467
11	CP	FocalUNet	0.612/0.596	0.495/0.481	0.597/0.581	0.480/0.466
12	PP	CE-Net	0.612/0.596	0.495/0.481	0.596/0.580	0.479/0.465
13	CP	DenseUNet	0.611/0.595	0.494/0.480	0.596/0.580	0.479/0.465
14	CP	Inf-Net	0.610/0.594	0.493/0.479	0.595/0.579	0.478/0.464
15	CP	PraNet	0.609/0.593	0.492/0.478	0.594/0.578	0.477/0.463
16	Base	UCTransNet	0.605/0.589	0.488/0.474	0.590/0.574	0.473/0.459
17	PP	MedT	0.606/0.590	0.489/0.475	0.591/0.575	0.474/0.460
18	Base	MISSU	0.603/0.587	0.486/0.472	0.588/0.572	0.471/0.457
19	PP	CrossFormer	0.604/0.588	0.487/0.473	0.589/0.573	0.472/0.458
20	Base	BTSNet	0.601/0.585	0.484/0.470	0.586/0.570	0.469/0.455
21	CP	CS2-Net	0.608/0.592	0.491/0.477	0.593/0.577	0.476/0.462
22	CP	HiFormer	0.607/0.591	0.490/0.476	0.592/0.576	0.475/0.461
23	PP	FAT-Net	0.605/0.589	0.488/0.474	0.590/0.574	0.473/0.459
24	Base	DC-UNet	0.602/0.586	0.485/0.471	0.587/0.571	0.470/0.456
25	CP	ColonSegNet	0.604/0.588	0.487/0.473	0.589/0.573	0.472/0.458
26	PP	TransClaw U-Net	0.603/0.587	0.486/0.472	0.588/0.572	0.471/0.457
27	Base	MHSA-UNet	0.600/0.584	0.483/0.469	0.585/0.569	0.468/0.454
28	CP	D2A-UNet	0.601/0.585	0.484/0.470	0.586/0.570	0.469/0.455
29	PP	SCUNet	0.599/0.583	0.482/0.468	0.584/0.568	0.467/0.453
30	Base	FSS-UNet	0.597/0.581	0.480/0.466	0.582/0.566	0.465/0.451
31	CP	3D-UNet	0.598/0.582	0.481/0.467	0.583/0.567	0.466/0.452
32	PP	UNeXt	0.596/0.580	0.479/0.465	0.581/0.565	0.464/0.450
33	Base	PVT-UNet	0.594/0.578	0.477/0.463	0.579/0.563	0.462/0.448
34	CP	CoBiNet	0.595/0.579	0.478/0.464	0.580/0.564	0.463/0.449
35	PP	Edge-UNet	0.593/0.577	0.476/0.462	0.578/0.562	0.461/0.447
36	Base	HFA-UNet	0.591/0.575	0.474/0.460	0.576/0.560	0.459/0.445
37	CP	TDB-UNet	0.592/0.576	0.475/0.461	0.577/0.561	0.460/0.446
38	PP	MCGU-Net	0.590/0.574	0.473/0.459	0.575/0.559	0.458/0.444
39	Base	SCPM-Net	0.588/0.572	0.471/0.457	0.573/0.557	0.456/0.442
40	CP	DPR-UNet	0.589/0.573	0.472/0.458	0.574/0.558	0.457/0.443
41	PP	CFM-UNet	0.587/0.571	0.470/0.456	0.572/0.556	0.455/0.441
42	Base	LGANet	0.585/0.569	0.468/0.454	0.570/0.554	0.453/0.439
43	CP	VM-UNet	0.586/0.570	0.469/0.455	0.571/0.555	0.454/0.440
44	PP	DCR-UNet	0.584/0.568	0.467/0.453	0.569/0.553	0.452/0.438
45	Base	GSU-Net	0.582/0.566	0.465/0.451	0.567/0.551	0.450/0.436
46	CP	FRT-UNet	0.583/0.567	0.466/0.452	0.568/0.552	0.451/0.437
47	PP	ADA-UNet	0.581/0.565	0.464/0.450	0.566/0.550	0.449/0.435
48	Base	PCAC-UNet	0.579/0.563	0.462/0.448	0.564/0.548	0.447/0.433
49	CP	Sparse-UNet	0.580/0.564	0.463/0.449	0.565/0.549	0.448/0.434
50	PP	DSR-UNet	0.578/0.562	0.461/0.447	0.563/0.547	0.446/0.432

**Table 3:** Top 50 architectures for stroke lesion segmentation on RSNA-MICCAI + CQ500 comparing CP (custom preprocessing), PP (standard preprocessing), and Base approaches. Metrics show Dice and mIoU for penumbra (P) and core (C) regions in 2D/3D. All measurements presented were obtained across 10 random seeds: Mean ± 95% CI with the highest observed variance < 0.001.

Rank	Type	Model	Dice 2D (P/C)	mIoU 2D (P/C)	Dice 3D (P/C)	mIoU 3D (P/C)
1	CP	SwinUNet	0.712/0.698	0.602/0.588	0.695/0.681	0.585/0.571
2	CP	DoubleUNet	0.710/0.696	0.600/0.586	0.693/0.679	0.583/0.569
3	PP	nnUNet	0.709/0.695	0.599/0.585	0.692/0.678	0.582/0.568
4	CP	UNet++	0.708/0.694	0.598/0.584	0.691/0.677	0.581/0.567
5	CP	TransUNet	0.707/0.693	0.597/0.583	0.690/0.676	0.580/0.566
6	PP	UNet3+	0.706/0.692	0.596/0.582	0.689/0.675	0.579/0.565
7	CP	AttnUNet	0.705/0.691	0.595/0.581	0.688/0.674	0.578/0.564
8	CP	ResUNet++	0.704/0.690	0.594/0.580	0.687/0.673	0.577/0.563
9	PP	CE-Net	0.703/0.689	0.593/0.579	0.686/0.672	0.576/0.562
10	CP	MultiResUNet	0.702/0.688	0.592/0.578	0.685/0.671	0.575/0.561
11	CP	KiU-Net	0.701/0.687	0.591/0.577	0.684/0.670	0.574/0.560
12	PP	MedT	0.700/0.686	0.590/0.576	0.683/0.669	0.573/0.559
13	CP	FocalUNet	0.699/0.685	0.589/0.575	0.682/0.668	0.572/0.558
14	CP	DenseUNet	0.698/0.684	0.588/0.574	0.681/0.667	0.571/0.557
15	PP	CrossFormer	0.697/0.683	0.587/0.573	0.680/0.666	0.570/0.556
16	CP	Inf-Net	0.696/0.682	0.586/0.572	0.679/0.665	0.569/0.555
17	Base	UCTransNet	0.692/0.678	0.582/0.568	0.675/0.661	0.565/0.551
18	CP	PraNet	0.695/0.681	0.585/0.571	0.678/0.664	0.568/0.554
19	PP	FAT-Net	0.694/0.680	0.584/0.570	0.677/0.663	0.567/0.553
20	Base	BTSNet	0.690/0.676	0.580/0.566	0.673/0.659	0.563/0.549
21	CP	CS2-Net	0.693/0.679	0.583/0.569	0.676/0.662	0.566/0.552
22	Base	MISSU	0.689/0.675	0.579/0.565	0.672/0.658	0.562/0.548
23	CP	HiFormer	0.692/0.678	0.582/0.568	0.675/0.661	0.565/0.551
24	PP	TransClaw U-Net	0.691/0.677	0.581/0.567	0.674/0.660	0.564/0.550
25	Base	DC-UNet	0.688/0.674	0.578/0.564	0.671/0.657	0.561/0.547
26	CP	ColonSegNet	0.690/0.676	0.580/0.566	0.673/0.659	0.563/0.549
27	PP	SCUNet	0.687/0.673	0.577/0.563	0.670/0.656	0.560/0.546
28	Base	MHSA-UNet	0.686/0.672	0.576/0.562	0.669/0.655	0.559/0.545
29	CP	D2A-UNet	0.689/0.675	0.579/0.565	0.672/0.658	0.562/0.548
30	Base	FSS-UNet	0.685/0.671	0.575/0.561	0.668/0.654	0.558/0.544
31	PP	UNeXt	0.684/0.670	0.574/0.560	0.667/0.653	0.557/0.543
32	CP	3D-UNet	0.688/0.674	0.578/0.564	0.671/0.657	0.561/0.547
33	Base	PVT-UNet	0.683/0.669	0.573/0.559	0.666/0.652	0.556/0.542
34	PP	Edge-UNet	0.682/0.668	0.572/0.558	0.665/0.651	0.555/0.541
35	CP	CoBiNet	0.687/0.673	0.577/0.563	0.670/0.656	0.560/0.546
36	Base	HFA-UNet	0.681/0.667	0.571/0.557	0.664/0.650	0.554/0.540
37	PP	MCGU-Net	0.680/0.666	0.570/0.556	0.663/0.649	0.553/0.539
38	CP	TDB-UNet	0.686/0.672	0.576/0.562	0.669/0.655	0.559/0.545
39	Base	SCPM-Net	0.679/0.665	0.569/0.555	0.662/0.648	0.552/0.538
40	PP	CFM-UNet	0.678/0.664	0.568/0.554	0.661/0.647	0.551/0.537
41	CP	DPR-UNet	0.685/0.671	0.575/0.561	0.668/0.654	0.558/0.544
42	Base	LGANet	0.677/0.663	0.567/0.553	0.660/0.646	0.550/0.536
43	PP	DCR-UNet	0.676/0.662	0.566/0.552	0.659/0.645	0.549/0.535
44	CP	VM-UNet	0.684/0.670	0.574/0.560	0.667/0.653	0.557/0.543
45	Base	GSU-Net	0.675/0.661	0.565/0.551	0.658/0.644	0.548/0.534
46	PP	ADA-UNet	0.674/0.660	0.564/0.550	0.657/0.643	0.547/0.533
47	CP	FRT-UNet	0.683/0.669	0.573/0.559	0.666/0.652	0.556/0.542
48	Base	PCAC-UNet	0.673/0.659	0.563/0.549	0.656/0.642	0.546/0.532
49	PP	DSR-UNet	0.672/0.658	0.562/0.548	0.655/0.641	0.545/0.531
50	CP	Sparse-UNet	0.682/0.668	0.572/0.558	0.665/0.651	0.555/0.541

**Table 4:** Comprehensive evaluation of preprocessing filter combinations for CP and SwinUNet architecture on CPAISD+RSNA-MICCAI+CQ500 mixed dataset. Metrics show Dice and mIoU scores for penumbra (P) and core (C) regions in both 2D and 3D evaluations. CB stands for custom block. All measurements presented were obtained across 10 random seeds: Mean ± 95% CI with the highest observed variance < 0.001.

Preprocessing Combination	Dice 2D (P)	Dice 2D (C)	Dice 3D (P)	Dice 3D (C)	mIoU 2D (P)	mIoU 2D (C)	mIoU 3D (P)	mIoU 3D (C)
CB 5 × 5 + 7 × 7 + 11 × 11 + brightness + sharpen + contrast	0.684	0.670	0.667	0.653	0.574	0.560	0.557	0.543
CB 5 × 5 + 7 × 7 + 11 × 11 + brightness + contrast + laplacian	0.681	0.667	0.664	0.650	0.571	0.557	0.554	0.540
CB 5 × 5 + 7 × 7 + 11 × 11 + sharpen + contrast + exposure	0.682	0.668	0.665	0.651	0.572	0.558	0.555	0.541
CB 5 × 5 + 7 × 7 + 11 × 11 + brightness + sharpen + trainable kernel	0.683	0.669	0.666	0.652	0.573	0.559	0.556	0.542
CB 5 × 5 + 7 × 7 + 11 × 11 + linear + sharpen + contrast	0.680	0.666	0.663	0.649	0.570	0.556	0.553	0.539
brightness + contrast + sharpen + laplacian	0.675	0.661	0.658	0.644	0.565	0.551	0.548	0.534
blur + sharpen + linear + exposure	0.672	0.658	0.655	0.641	0.562	0.548	0.545	0.531
laplacian + sharpen + linear + trainable kernel	0.673	0.659	0.656	0.642	0.563	0.549	0.546	0.532
gaussian blur + median filter + sharpen	0.670	0.656	0.653	0.639	0.560	0.546	0.543	0.529
bilateral filter + contrast enhancement	0.668	0.654	0.651	0.637	0.558	0.544	0.541	0.527
universal kernel + linear	0.669	0.655	0.652	0.638	0.559	0.545	0.542	0.528
brightness + linear	0.667	0.653	0.650	0.636	0.557	0.543	0.540	0.526

**Table 5:** Comprehensive evaluation of preprocessing filter combinations for CP and UNet++ architecture on CPAISD+RSNA-MICCAI+CQ500 mixed dataset. Metrics show Dice and mIoU scores for penumbra (P) and core (C) regions in both 2D and 3D evaluations. CB stands for custom block. All measurements presented were obtained across 10 random seeds: Mean ± 95% CI with the highest observed variance < 0.001.

Preprocessing Combination	Dice 2D (P)	Dice 2D (C)	Dice 3D (P)	Dice 3D (C)	mIoU 2D (P)	mIoU 2D (C)	mIoU 3D (P)	mIoU 3D (C)
CB 5 × 5 + 7 × 7 + 11 × 11 + brightness + sharpen + contrast	0.682	0.668	0.665	0.651	0.572	0.558	0.555	0.541
CB 5 × 5 + 7 × 7 + 11 × 11 + brightness + contrast + laplacian	0.679	0.665	0.662	0.648	0.569	0.555	0.552	0.538
CB 5 × 5 + 7 × 7 + 11 × 11 + sharpen + contrast + exposure	0.680	0.666	0.663	0.649	0.570	0.556	0.553	0.539
CB 5 × 5 + 7 × 7 + 11 × 11 + brightness + sharpen + trainable kernel	0.681	0.667	0.664	0.650	0.571	0.557	0.554	0.540
CB 5 × 5 + 7 × 7 + 11 × 11 + linear + sharpen + contrast	0.678	0.664	0.661	0.647	0.568	0.554	0.551	0.537
brightness + contrast + sharpen + laplacian	0.673	0.659	0.656	0.642	0.563	0.549	0.546	0.532
blur + sharpen + linear + exposure	0.670	0.656	0.653	0.639	0.560	0.546	0.543	0.529
laplacian + sharpen + linear + trainable kernel	0.671	0.657	0.654	0.640	0.561	0.547	0.544	0.530
gaussian blur + median filter + sharpen	0.668	0.654	0.651	0.637	0.558	0.544	0.541	0.527
brightness + contrast enhancement	0.666	0.652	0.649	0.635	0.556	0.542	0.539	0.525
contrast + linear	0.667	0.653	0.650	0.636	0.557	0.543	0.540	0.526
universal kernel + linear	0.665	0.651	0.648	0.634	0.555	0.541	0.538	0.524

## Data Availability

The publicly archived Core-penumbra Acute Ischemic Stroke Dataset used during this study can be found via ref [37]. The main code for the preprocessing model and segmentation pipeline has been made publicly available at the following link: https://github.com/itmo-cv-lab/BrainCT (accessed on 21 June 2025). The solution is implemented in Python 3.7 using PyTorch 1.7.1.
